# [μ-*N*,*N*,*N*′,*N*′-Tetra­kis(2-pyridyl­meth­yl)butane-1,4-diamine]­bis­[diacetato­cadmium(II)] nona­hydrate

**DOI:** 10.1107/S1600536810034550

**Published:** 2010-09-04

**Authors:** Mark Bartholomä, Hoi Cheung, Jon Zubieta

**Affiliations:** a Department of Chemistry, Syracuse University, Syracuse, New York 13244, USA

## Abstract

The title dinuclear complex, [Cd_2_(CH_3_CO_2_)_4_(C_28_H_32_N_6_)]·9H_2_O, is located on a crystallographic inversion center. The unique Cd^II^ ion displays a 5 + 2 coordination. A distorted square-pyramidal geometry is formed by the dipicolyl­amine unit of the ligand *via* the N atoms in a meridional fashion and two O atoms of the acetate ligands with short Cd—O distances. The coordination is completed by two loosely bound O atoms of the acetate ligands. The Cd—N distances involving the pyridine N atoms differ slightly from each other and the Cd—N distance involving the tertiary N atom is the longest. In the crystal structure, complex mol­ecules and solvent water mol­ecules are connected into a three-dimensional network *via* inter­molecular O—H⋯O hydrogen bonds. One of the water mol­ecules lies on a twofold rotation axis.

## Related literature

For related crystal structures of tetra­kis­(pyridin-2-yl-meth­yl)alkyl-diamine compounds, see: Fujihara *et al.* (2004 ▶); Mambanda *et al.* (2007 ▶). For dinuclear platinum complexes of similar ligands, see: Ertürk *et al.* (2007 ▶). For the superoxide dismutase activity of iron complexes, see: Tamura *et al.* (2000 ▶). For the use of the dipicolyl­amine moiety for binding of the *M*(CO)_3_ core (*M* = Re, ^99*m*^Tc), see: Bartholomä *et al.* (2009 ▶). For crystal structures closely related to the title compound, see: Bartholomä *et al.* (2010*a* ▶,*b* ▶,*c* ▶,*d* ▶).
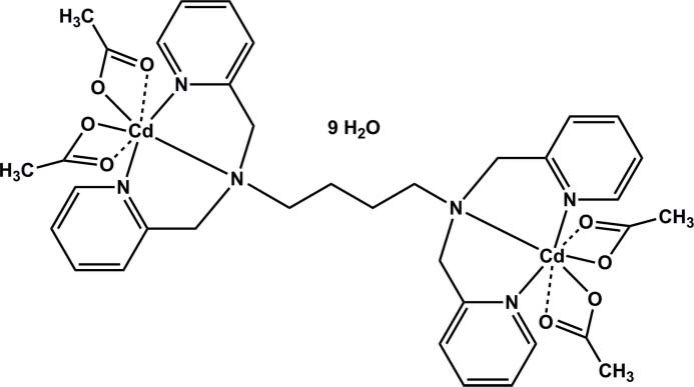


## Experimental

### 

#### Crystal data

[Cd_2_(C_2_H_3_O_2_)_4_(C_28_H_32_N_6_)]·9H_2_O
*M* *_r_* = 1075.72
Monoclinic, 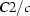

*a* = 15.9680 (17) Å
*b* = 11.4320 (12) Å
*c* = 26.451 (3) Å
β = 100.127 (2)°
*V* = 4753.3 (9) Å^3^
*Z* = 4
Mo *K*α radiation
μ = 0.97 mm^−1^
*T* = 90 K
0.30 × 0.20 × 0.10 mm

#### Data collection

Bruker SMART APEX diffractometer
Absorption correction: multi-scan (*SADABS*; Bruker, 1998 ▶) *T*_min_ = 0.760, *T*_max_ = 0.910
23441 measured reflections
5847 independent reflections
5621 reflections with *I* > 2σ(*I*)
*R*_int_ = 0.022

#### Refinement

*R*[*F* ^2^ > 2σ(*F* ^2^)] = 0.045
*wR*(*F* ^2^) = 0.103
*S* = 1.20
5847 reflections
314 parameters
H atoms treated by a mixture of independent and constrained refinement
Δρ_max_ = 1.55 e Å^−3^
Δρ_min_ = −0.57 e Å^−3^

### 

Data collection: *SMART* (Bruker, 1998 ▶); cell refinement: *SAINT* (Bruker, 1998 ▶); data reduction: *SAINT*; program(s) used to solve structure: *SHELXS97* (Sheldrick, 2008 ▶); program(s) used to refine structure: *SHELXL97* (Sheldrick, 2008 ▶); molecular graphics: *DIAMOND* (Brandenburg & Putz, 1999 ▶); software used to prepare material for publication: *SHELXTL* (Sheldrick, 2008 ▶).

## Supplementary Material

Crystal structure: contains datablocks I, global. DOI: 10.1107/S1600536810034550/lh5104sup1.cif

Structure factors: contains datablocks I. DOI: 10.1107/S1600536810034550/lh5104Isup2.hkl

Additional supplementary materials:  crystallographic information; 3D view; checkCIF report

## Figures and Tables

**Table 1:** Selected bond lengths (Å)

Cd1—O4	2.240 (2)
Cd1—O2	2.251 (3)
Cd1—N3	2.313 (3)
Cd1—N2	2.379 (3)
Cd1—N1	2.405 (3)
Cd1—O1	2.550 (3)
Cd1—O3	2.729 (4)

**Table 2:** Hydrogen-bond geometry (Å, °)

*D*—H⋯*A*	*D*—H	H⋯*A*	*D*⋯*A*	*D*—H⋯*A*
O9—H9*A*⋯O3	0.80 (6)	2.06 (6)	2.829 (5)	160 (6)
O6—H6*A*⋯O4	0.77 (5)	1.98 (5)	2.745 (4)	170 (5)
O9—H9*B*⋯O5^i^	0.79 (5)	2.07 (5)	2.852 (3)	170 (5)
O7—H7*C*⋯O1^ii^	0.76 (5)	1.91 (5)	2.673 (4)	177 (5)
O8—H8*A*⋯O9^iii^	0.73 (5)	2.04 (5)	2.769 (4)	172 (5)
O6—H6*B*⋯O8^iv^	0.85 (5)	1.97 (5)	2.792 (4)	164 (5)
O5—H5*A*⋯O7^ii^	0.76 (4)	1.96 (4)	2.708 (3)	170 (5)
O8—H8*B*⋯O6^v^	0.78 (5)	2.06 (5)	2.825 (4)	166 (4)

Symmetry codes: (i) 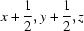; (ii) 
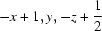
; (iii) 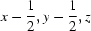; (iv) 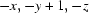; (v) 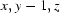.

**Fig. 1.**
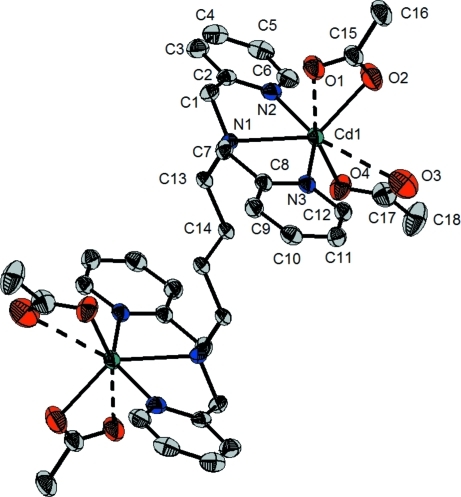
The crystal structure of the title complex. The displacement ellipsoids are drawn at 50% probability level. Water of crystallization and hydrogen atoms are omitted for clarity. Unlabeled atoms are related by the symmetry code (-x, -y+1, -z).

## supplementary crystallographic information

### Comment

The described ligand *N^1^*,*N^1^*,*N^4^*,*N^4^*-tetrakis(pyridin-2-ylmethyl)butane-1,4-diamine has been used as starting material in the hydrothermal synthesis of metal-organic transition metal/molybdateoxide frameworks in the principal author's laboratory. The dipicolylamine moiety has originally been used in our laboratory as metal chelating entity for binding of the *M*(CO)_3_ core (*M* = Re,^99^*^m^*Tc) for radiopharmaceutical purposes. However, a different coordination mode has been observed for the *M*(CO)_3_ core in which the dipicolylamine metal chelate is bound in a facial manner (Bartholomä, 2009).

Crystal structures of the ligands *N^1^*,*N^1^*,*N^3^*,*N^3^*-tetrakis(2-pyridiniomethyl)-1,3-diaminopropane and *N^1^*,*N^1^*,*N^4^*,*N^4^*-tetrakis(pyridin-2-ylmethyl)butane-1,4-diamine have been described recently (Fujihara, 2004; Mambanda, 2007). Superoxide dismutase activity of iron(II) complexes of *N^1^*,*N^1^*,*N^3^*,*N^3^*-tetrakis(2-pyridiniomethyl)-1,3-diaminopropane and related ligands has been investigated by Tamura *et al.* (2000). Studies on the thermodynamic and kinetic behaviour of the reaction of platinum(II) complexes of higher ligand homologues with chloride have been performed by Ertürk *et al.* (2007).

The title complex was prepared as part of a series with different cadmium and copper salts to study the coordination properties of the ligand with these metals without the interaction of metaloxide clusters (Bartholomä, 2010*b*,c,*d*). We have reported another crystal structure of a molecular dinuclear cadmium complex using the corresponding nitrate salt as metal source (Bartholomä, 2010*a*). In the cadmium nitrate structure, the Cd—N distances involving the pyridine N atoms [2.250 (2) Å and 2.251 (2) Å] are slightly shorter whereas the Cd—N distance involving the tertiary nitrogen atom [2.427 (2) Å] is marginally longer when compared to the related distances in the title compound.

### Experimental

*N^1^*,*N^1^*,*N^4^*,*N^4^*-tetrakis(pyridin-2-ylmethyl)butane-1,4-diamine. An amount of 1.00 g (11.34 mmol) 1,4-diaminobutane was dissolved in 30 ml anhydrous dichloroethane under an inert atmosphere (argon) followed by the addition of 4.55 ml (47.65 mmol) pyridine-2-carboxaldehyde. The mixture was stirred for 15 min at r.t. and then cooled with an ice bath prior to the portionwise addition of 14.43 g (68.06 mmol) sodium triacetoxyborohydride (gas evolution, exothermic reaction). The reaction was stirred overnight allowing the temperature slowly to rise to room temperature. The reaction was quenched by the dropwise addition of saturated sodium bicarbonate solution and stirring was continued until the gas evolution ceased. The mixture was separated and the organic layer was further washed with saturated sodium bicarbonate solution, water and brine. The organic phase was dried with anhydrous sodium sulfate, filtered and the solvent removed under reduced pressure. The crude reaction mixture was then purified by silica gel column chromatography starting with chloroform and increasing gradient to chloroform:methanol 10:1 (*v*/*v*). Yield: 4.02 g (78%). ^1^H NMR (CDCl_3_): *δ* = 8.40 (m, 4H), 7.51 (m, 4H), 7.39 (d, J = 7.81 Hz, 4H), 7.02 (m, 4H), 3.67 (s, 8H), 2.39 (m, 4H), 1.42 (m, 4H) p.p.m..

Synthesis of metal complex. To 2 ml of an aqueous solution of cadmium acetate, two equivalents (50 mg, 0.11 mmol) of *N^1^*,*N^1^*,*N^4^*,*N^4^*-tetrakis(pyridin-2-ylmethyl)butane-1,4-diamine in 2 ml methanol were added followed by the addition of 2 ml *N*,*N*-dimethylformamide. Single crystals were obtained after a week by slow evaporation of the solvents at room temperature.

### Refinement

All H atoms were placed in idealized positions and refined using a riding-model approximation with C—H(aryl) = 0.95Å, C—H(methyl) = 0.98Å and C—H (methylene) = 0.99Å and U_iso_(H) = 1.5U_eq_(C_methyl_) and 1.2U_eq_(C_methylene/aryl_). Water hydrogen atoms were located in a difference Fourier map and refined freely.

### Crystal data

**Table:**

[Cd_2_(C_2_H_3_O_2_)_4_(C_28_H_32_N_6_)]·9H_2_O	*F*(000) = 2208
*M**_r_* = 1075.72	*D*_x_ = 1.503 Mg m^−^^3^
Monoclinic, *C*2/*c*	Mo *K*α radiation, λ = 0.71073 Å
Hall symbol: -C 2yc	Cell parameters from 5663 reflections
*a* = 15.9680 (17) Å	θ = 2.6–28.3°
*b* = 11.4320 (12) Å	µ = 0.97 mm^−^^1^
*c* = 26.451 (3) Å	*T* = 90 K
β = 100.127 (2)°	Block, colourless
*V* = 4753.3 (9) Å^3^	0.30 × 0.20 × 0.10 mm
*Z* = 4	

### Data collection

**Table:**

Bruker SMART APEX diffractometer	5847 independent reflections
Radiation source: fine-focus sealed tube	5621 reflections with *I* > 2σ(*I*)
graphite	*R*_int_ = 0.022
Detector resolution: 512 pixels mm^-1^	θ_max_ = 28.3°, θ_min_ = 2.2°
φ and ω scans	*h* = −20→21
Absorption correction: multi-scan (*SADABS*; Bruker, 1998)	*k* = −15→15
*T*_min_ = 0.760, *T*_max_ = 0.910	*l* = −35→34
23441 measured reflections	

### Refinement

**Table:**

Refinement on *F*^2^	Primary atom site location: structure-invariant direct methods
Least-squares matrix: full	Secondary atom site location: difference Fourier map
*R*[*F*^2^ > 2σ(*F*^2^)] = 0.045	Hydrogen site location: inferred from neighbouring sites
*wR*(*F*^2^) = 0.103	H atoms treated by a mixture of independent and constrained refinement
*S* = 1.20	*w* = 1/[σ^2^(*F*_o_^2^) + (0.0355*P*)^2^ + 21.0991*P*] where *P* = (*F*_o_^2^ + 2*F*_c_^2^)/3
5847 reflections	(Δ/σ)_max_ = 0.001
314 parameters	Δρ_max_ = 1.55 e Å^−^^3^
0 restraints	Δρ_min_ = −0.56 e Å^−^^3^

### Special details

**Table:**

Geometry. All e.s.d.'s (except the e.s.d. in the dihedral angle between two l.s. planes) are estimated using the full covariance matrix. The cell e.s.d.'s are taken into account individually in the estimation of e.s.d.'s in distances, angles and torsion angles; correlations between e.s.d.'s in cell parameters are only used when they are defined by crystal symmetry. An approximate (isotropic) treatment of cell e.s.d.'s is used for estimating e.s.d.'s involving l.s. planes.
Refinement. Refinement of *F*^2^ against ALL reflections. The weighted *R*-factor *wR* and goodness of fit *S* are based on *F*^2^, conventional *R*-factors *R* are based on *F*, with *F* set to zero for negative *F*^2^. The threshold expression of *F*^2^ > σ(*F*^2^) is used only for calculating *R*-factors(gt) *etc*. and is not relevant to the choice of reflections for refinement. *R*-factors based on *F*^2^ are statistically about twice as large as those based on *F*, and *R*- factors based on ALL data will be even larger.

### Fractional atomic coordinates and isotropic or equivalent isotropic displacement parameters (Å^2^)

**Table:**

	*x*	*y*	*z*	*U*_iso_*/*U*_eq_	
Cd1	0.165978 (14)	0.604997 (19)	0.132379 (8)	0.02575 (8)	
O1	0.2134 (2)	0.4939 (3)	0.21610 (10)	0.0556 (9)	
O2	0.2542 (2)	0.6682 (3)	0.20295 (11)	0.0560 (8)	
O3	0.2906 (2)	0.7203 (3)	0.09388 (13)	0.0616 (9)	
O4	0.15464 (17)	0.7426 (2)	0.07125 (11)	0.0423 (6)	
O5	0.0000	0.2308 (3)	0.2500	0.0322 (7)	
O6	0.00239 (19)	0.8361 (3)	0.02426 (12)	0.0402 (6)	
O7	0.85601 (17)	0.3567 (3)	0.22061 (12)	0.0377 (6)	
O8	0.0253 (2)	0.0574 (3)	0.07224 (12)	0.0418 (6)	
O9	0.4442 (2)	0.6321 (3)	0.15079 (13)	0.0437 (7)	
N1	0.05268 (16)	0.4637 (2)	0.12010 (9)	0.0228 (5)	
N2	0.04401 (19)	0.6870 (3)	0.15915 (10)	0.0300 (6)	
N3	0.21759 (17)	0.4384 (2)	0.09883 (10)	0.0259 (5)	
C1	0.0067 (2)	0.4816 (3)	0.16377 (12)	0.0276 (6)	
H1A	0.0419	0.4517	0.1957	0.033*	
H1B	−0.0471	0.4366	0.1575	0.033*	
C2	−0.0127 (2)	0.6095 (3)	0.17043 (11)	0.0273 (6)	
C3	−0.0848 (2)	0.6442 (3)	0.18900 (13)	0.0349 (7)	
H3	−0.1241	0.5878	0.1971	0.042*	
C4	−0.0988 (3)	0.7620 (4)	0.19562 (14)	0.0407 (8)	
H4	−0.1474	0.7876	0.2088	0.049*	
C5	−0.0413 (3)	0.8423 (3)	0.18282 (13)	0.0396 (8)	
H5	−0.0500	0.9239	0.1864	0.047*	
C6	0.0289 (3)	0.8011 (3)	0.16482 (13)	0.0361 (8)	
H6	0.0686	0.8561	0.1560	0.043*	
C7	0.0917 (2)	0.3463 (3)	0.12297 (12)	0.0270 (6)	
H7A	0.0498	0.2899	0.1049	0.032*	
H7B	0.1056	0.3222	0.1594	0.032*	
C8	0.17205 (19)	0.3403 (3)	0.09967 (11)	0.0246 (6)	
C9	0.1976 (2)	0.2339 (3)	0.08241 (12)	0.0299 (7)	
H9	0.1638	0.1658	0.0832	0.036*	
C10	0.2736 (2)	0.2291 (3)	0.06395 (13)	0.0339 (7)	
H10	0.2928	0.1572	0.0521	0.041*	
C11	0.3211 (2)	0.3300 (3)	0.06300 (13)	0.0332 (7)	
H11	0.3733	0.3284	0.0505	0.040*	
C12	0.2915 (2)	0.4325 (3)	0.08031 (12)	0.0301 (7)	
H12	0.3240	0.5018	0.0793	0.036*	
C13	−0.00785 (19)	0.4817 (3)	0.07122 (11)	0.0264 (6)	
H13A	−0.0380	0.5567	0.0733	0.032*	
H13B	−0.0509	0.4185	0.0675	0.032*	
C14	0.03306 (18)	0.4836 (3)	0.02357 (11)	0.0246 (6)	
H14A	0.0571	0.4057	0.0184	0.029*	
H14B	0.0801	0.5413	0.0281	0.029*	
C15	0.2586 (2)	0.5762 (3)	0.23017 (13)	0.0387 (8)	
C16	0.3163 (3)	0.5767 (5)	0.28214 (16)	0.0603 (14)	
H16A	0.2953	0.5199	0.3047	0.090*	
H16B	0.3166	0.6550	0.2973	0.090*	
H16C	0.3742	0.5555	0.2780	0.090*	
C17	0.2281 (3)	0.7765 (4)	0.07178 (16)	0.0440 (9)	
C18	0.2414 (3)	0.8863 (4)	0.0423 (2)	0.0635 (14)	
H18A	0.2959	0.8813	0.0302	0.095*	
H18B	0.2419	0.9545	0.0648	0.095*	
H18C	0.1951	0.8944	0.0128	0.095*	
H8B	0.017 (3)	−0.008 (4)	0.0639 (16)	0.033 (11)*	
H5A	0.039 (2)	0.271 (4)	0.2553 (17)	0.035 (11)*	
H6B	0.001 (3)	0.859 (4)	−0.007 (2)	0.048 (13)*	
H8A	0.002 (3)	0.071 (4)	0.093 (2)	0.049 (15)*	
H7C	0.835 (3)	0.395 (4)	0.2383 (19)	0.048 (14)*	
H9B	0.463 (3)	0.653 (4)	0.1789 (19)	0.043 (13)*	
H6A	0.047 (3)	0.812 (4)	0.0345 (19)	0.052 (15)*	
H7D	0.824 (3)	0.309 (5)	0.2106 (19)	0.054 (15)*	
H9A	0.400 (4)	0.665 (5)	0.141 (2)	0.069 (18)*	

### Atomic displacement parameters (Å^2^)

**Table:**

	*U*^11^	*U*^22^	*U*^33^	*U*^12^	*U*^13^	*U*^23^
Cd1	0.02604 (13)	0.02573 (12)	0.02352 (12)	−0.00736 (8)	−0.00101 (8)	0.00419 (8)
O1	0.0564 (18)	0.072 (2)	0.0323 (14)	−0.0304 (16)	−0.0097 (12)	0.0150 (14)
O2	0.0591 (19)	0.0571 (19)	0.0434 (16)	−0.0261 (15)	−0.0137 (14)	0.0117 (14)
O3	0.0560 (19)	0.065 (2)	0.0574 (19)	0.0113 (16)	−0.0089 (15)	0.0048 (16)
O4	0.0395 (14)	0.0400 (14)	0.0471 (15)	−0.0045 (11)	0.0070 (12)	0.0182 (12)
O5	0.0285 (18)	0.0317 (18)	0.0355 (18)	0.000	0.0031 (15)	0.000
O6	0.0330 (15)	0.0471 (16)	0.0420 (16)	0.0024 (12)	0.0104 (12)	0.0054 (13)
O7	0.0262 (13)	0.0366 (14)	0.0509 (16)	−0.0037 (11)	0.0086 (12)	−0.0113 (12)
O8	0.0517 (17)	0.0372 (16)	0.0395 (15)	−0.0072 (13)	0.0165 (13)	−0.0059 (12)
O9	0.0422 (16)	0.0450 (16)	0.0430 (17)	0.0027 (13)	0.0049 (13)	−0.0166 (13)
N1	0.0230 (12)	0.0252 (12)	0.0198 (11)	−0.0018 (10)	0.0026 (9)	0.0023 (9)
N2	0.0377 (15)	0.0313 (14)	0.0202 (12)	−0.0047 (12)	0.0033 (11)	0.0014 (10)
N3	0.0250 (13)	0.0292 (13)	0.0209 (12)	−0.0040 (10)	−0.0025 (10)	0.0043 (10)
C1	0.0308 (16)	0.0300 (16)	0.0225 (14)	−0.0035 (13)	0.0062 (12)	0.0055 (12)
C2	0.0330 (16)	0.0311 (16)	0.0172 (13)	−0.0019 (13)	0.0022 (11)	0.0031 (11)
C3	0.0371 (18)	0.0418 (19)	0.0265 (16)	0.0007 (15)	0.0072 (14)	0.0027 (14)
C4	0.045 (2)	0.047 (2)	0.0302 (17)	0.0088 (17)	0.0070 (15)	−0.0028 (15)
C5	0.055 (2)	0.0332 (18)	0.0279 (17)	0.0057 (16)	0.0014 (16)	−0.0036 (14)
C6	0.048 (2)	0.0319 (17)	0.0264 (16)	−0.0060 (15)	0.0022 (14)	−0.0021 (13)
C7	0.0287 (15)	0.0219 (14)	0.0302 (15)	−0.0033 (12)	0.0047 (12)	0.0038 (12)
C8	0.0237 (14)	0.0273 (15)	0.0210 (13)	−0.0030 (12)	−0.0014 (11)	0.0036 (11)
C9	0.0296 (16)	0.0307 (16)	0.0280 (15)	−0.0045 (13)	0.0009 (12)	0.0026 (12)
C10	0.0331 (17)	0.0369 (18)	0.0294 (16)	0.0033 (14)	−0.0007 (13)	−0.0011 (14)
C11	0.0255 (16)	0.0435 (19)	0.0298 (16)	−0.0024 (14)	0.0027 (13)	0.0017 (14)
C12	0.0246 (15)	0.0388 (17)	0.0250 (15)	−0.0062 (13)	−0.0005 (12)	0.0039 (13)
C13	0.0232 (14)	0.0348 (16)	0.0195 (13)	−0.0054 (12)	−0.0009 (11)	0.0000 (12)
C14	0.0211 (14)	0.0299 (15)	0.0211 (14)	−0.0029 (12)	−0.0007 (11)	−0.0003 (11)
C15	0.041 (2)	0.045 (2)	0.0262 (16)	−0.0158 (16)	−0.0051 (14)	0.0073 (14)
C16	0.056 (3)	0.081 (3)	0.035 (2)	−0.033 (2)	−0.0160 (19)	0.016 (2)
C17	0.047 (2)	0.041 (2)	0.041 (2)	−0.0010 (17)	−0.0010 (17)	0.0084 (16)
C18	0.055 (3)	0.053 (3)	0.085 (4)	−0.008 (2)	0.019 (3)	0.030 (3)

### Geometric parameters (Å, °)

**Table:**

Cd1—O4	2.240 (2)	C3—H3	0.9500
Cd1—O4	2.240 (2)	C4—C5	1.382 (6)
Cd1—O2	2.251 (3)	C4—H4	0.9500
Cd1—N3	2.313 (3)	C5—C6	1.376 (6)
Cd1—N2	2.379 (3)	C5—H5	0.9500
Cd1—N1	2.405 (3)	C6—H6	0.9500
Cd1—O1	2.550 (3)	C7—C8	1.519 (4)
Cd1—O3	2.729 (4)	C7—H7A	0.9900
O1—C15	1.205 (5)	C7—H7B	0.9900
O2—C15	1.269 (5)	C8—C9	1.386 (5)
O3—C17	1.242 (5)	C9—C10	1.386 (5)
O4—C17	1.233 (5)	C9—H9	0.9500
O5—H5A	0.76 (4)	C10—C11	1.384 (5)
O6—H6B	0.85 (5)	C10—H10	0.9500
O6—H6A	0.77 (5)	C11—C12	1.371 (5)
O7—H7C	0.76 (5)	C11—H11	0.9500
O7—H7D	0.77 (5)	C12—H12	0.9500
O8—H8B	0.78 (5)	C13—C14	1.518 (4)
O8—H8A	0.73 (5)	C13—H13A	0.9900
O9—H9B	0.79 (5)	C13—H13B	0.9900
O9—H9A	0.80 (6)	C14—C14^i^	1.532 (6)
N1—C7	1.475 (4)	C14—H14A	0.9900
N1—C13	1.486 (4)	C14—H14B	0.9900
N1—C1	1.488 (4)	C15—C16	1.514 (5)
N2—C2	1.338 (4)	C16—H16A	0.9800
N2—C6	1.340 (5)	C16—H16B	0.9800
N3—C8	1.339 (4)	C16—H16C	0.9800
N3—C12	1.357 (4)	C17—O4	1.233 (5)
C1—C2	1.512 (5)	C17—O3	1.242 (5)
C1—H1A	0.9900	C17—C18	1.513 (6)
C1—H1B	0.9900	C18—H18A	0.9800
C2—C3	1.387 (5)	C18—H18B	0.9800
C3—C4	1.381 (5)	C18—H18C	0.9800
			
O4—Cd1—O4	0.0 (2)	C6—C5—C4	118.3 (3)
O4—Cd1—O2	109.44 (11)	C6—C5—H5	120.8
O4—Cd1—O2	109.44 (11)	C4—C5—H5	120.8
O4—Cd1—N3	106.87 (10)	N2—C6—C5	123.1 (4)
O4—Cd1—N3	106.87 (10)	N2—C6—H6	118.5
O2—Cd1—N3	111.66 (11)	C5—C6—H6	118.5
O4—Cd1—N2	88.38 (10)	N1—C7—C8	113.6 (2)
O4—Cd1—N2	88.38 (10)	N1—C7—H7A	108.9
O2—Cd1—N2	93.01 (12)	C8—C7—H7A	108.9
N3—Cd1—N2	143.54 (9)	N1—C7—H7B	108.9
O4—Cd1—N1	114.26 (9)	C8—C7—H7B	108.9
O4—Cd1—N1	114.26 (9)	H7A—C7—H7B	107.7
O2—Cd1—N1	132.37 (10)	N3—C8—C9	122.5 (3)
N3—Cd1—N1	72.86 (9)	N3—C8—C7	117.9 (3)
N2—Cd1—N1	70.68 (9)	C9—C8—C7	119.5 (3)
O4—Cd1—O1	161.92 (10)	C8—C9—C10	118.5 (3)
O4—Cd1—O1	161.92 (10)	C8—C9—H9	120.7
O2—Cd1—O1	52.59 (10)	C10—C9—H9	120.7
N3—Cd1—O1	81.46 (10)	C11—C10—C9	119.3 (3)
N2—Cd1—O1	94.11 (10)	C11—C10—H10	120.3
N1—Cd1—O1	83.35 (9)	C9—C10—H10	120.3
O4—Cd1—O3	50.44 (9)	C12—C11—C10	119.0 (3)
O4—Cd1—O3	50.44 (9)	C12—C11—H11	120.5
O2—Cd1—O3	76.31 (11)	C10—C11—H11	120.5
N3—Cd1—O3	85.54 (10)	N3—C12—C11	122.4 (3)
N2—Cd1—O3	127.56 (10)	N3—C12—H12	118.8
N1—Cd1—O3	148.74 (9)	C11—C12—H12	118.8
O1—Cd1—O3	116.10 (9)	N1—C13—C14	114.5 (2)
C15—O1—Cd1	87.1 (2)	N1—C13—H13A	108.6
C15—O2—Cd1	99.7 (2)	C14—C13—H13A	108.6
C17—O3—Cd1	81.2 (3)	N1—C13—H13B	108.6
C17—O4—Cd1	104.9 (2)	C14—C13—H13B	108.6
H6B—O6—H6A	108 (5)	H13A—C13—H13B	107.6
H7C—O7—H7D	106 (5)	C13—C14—C14^i^	110.1 (3)
H8B—O8—H8A	110 (5)	C13—C14—H14A	109.6
H9B—O9—H9A	109 (5)	C14^i^—C14—H14A	109.6
C7—N1—C13	112.0 (2)	C13—C14—H14B	109.6
C7—N1—C1	110.3 (2)	C14^i^—C14—H14B	109.6
C13—N1—C1	108.8 (2)	H14A—C14—H14B	108.2
C7—N1—Cd1	107.62 (18)	O1—C15—O2	120.1 (3)
C13—N1—Cd1	112.52 (18)	O1—C15—C16	121.2 (3)
C1—N1—Cd1	105.36 (18)	O2—C15—C16	118.5 (3)
C2—N2—C6	118.6 (3)	C15—C16—H16A	109.5
C2—N2—Cd1	115.3 (2)	C15—C16—H16B	109.5
C6—N2—Cd1	126.1 (2)	H16A—C16—H16B	109.5
C8—N3—C12	118.3 (3)	C15—C16—H16C	109.5
C8—N3—Cd1	116.9 (2)	H16A—C16—H16C	109.5
C12—N3—Cd1	124.8 (2)	H16B—C16—H16C	109.5
N1—C1—C2	111.3 (2)	O4—C17—O3	121.8 (4)
N1—C1—H1A	109.4	O4—C17—O3	121.8 (4)
C2—C1—H1A	109.4	O4—C17—O3	121.8 (4)
N1—C1—H1B	109.4	O4—C17—O3	121.8 (4)
C2—C1—H1B	109.4	O4—C17—C18	118.3 (4)
H1A—C1—H1B	108.0	O4—C17—C18	118.3 (4)
N2—C2—C3	121.7 (3)	O3—C17—C18	119.8 (4)
N2—C2—C1	117.0 (3)	O3—C17—C18	119.8 (4)
C3—C2—C1	121.2 (3)	C17—C18—H18A	109.5
C4—C3—C2	119.2 (4)	C17—C18—H18B	109.5
C4—C3—H3	120.4	H18A—C18—H18B	109.5
C2—C3—H3	120.4	C17—C18—H18C	109.5
C3—C4—C5	119.1 (4)	H18A—C18—H18C	109.5
C3—C4—H4	120.4	H18B—C18—H18C	109.5
C5—C4—H4	120.4		
			
O4—Cd1—O1—C15	2.7 (5)	N3—Cd1—N2—C6	−160.9 (2)
O4—Cd1—O1—C15	2.7 (5)	N1—Cd1—N2—C6	−160.6 (3)
O2—Cd1—O1—C15	−4.0 (3)	O1—Cd1—N2—C6	117.9 (3)
N3—Cd1—O1—C15	121.8 (3)	O3—Cd1—N2—C6	−9.8 (3)
N2—Cd1—O1—C15	−94.7 (3)	O4—Cd1—N3—C8	−125.3 (2)
N1—Cd1—O1—C15	−164.7 (3)	O4—Cd1—N3—C8	−125.3 (2)
O3—Cd1—O1—C15	41.0 (3)	O2—Cd1—N3—C8	115.0 (2)
O4—Cd1—O2—C15	−173.9 (3)	N2—Cd1—N3—C8	−14.2 (3)
O4—Cd1—O2—C15	−173.9 (3)	N1—Cd1—N3—C8	−14.5 (2)
N3—Cd1—O2—C15	−55.8 (3)	O1—Cd1—N3—C8	71.1 (2)
N2—Cd1—O2—C15	96.7 (3)	O3—Cd1—N3—C8	−171.6 (2)
N1—Cd1—O2—C15	30.4 (3)	O4—Cd1—N3—C12	57.2 (3)
O1—Cd1—O2—C15	3.9 (3)	O4—Cd1—N3—C12	57.2 (3)
O3—Cd1—O2—C15	−135.3 (3)	O2—Cd1—N3—C12	−62.5 (3)
O4—Cd1—O3—O3	0.00 (18)	N2—Cd1—N3—C12	168.3 (2)
O4—Cd1—O3—O3	0.00 (18)	N1—Cd1—N3—C12	168.0 (3)
O2—Cd1—O3—O3	0.00 (11)	O1—Cd1—N3—C12	−106.4 (2)
N3—Cd1—O3—O3	0.00 (13)	O3—Cd1—N3—C12	10.9 (2)
N2—Cd1—O3—O3	0.00 (13)	C7—N1—C1—C2	164.2 (3)
N1—Cd1—O3—O3	0.00 (6)	C13—N1—C1—C2	−72.5 (3)
O1—Cd1—O3—O3	0.00 (16)	Cd1—N1—C1—C2	48.4 (3)
O4—Cd1—O3—C17	7.3 (2)	C6—N2—C2—C3	−1.8 (5)
O4—Cd1—O3—C17	7.3 (2)	Cd1—N2—C2—C3	176.7 (2)
O2—Cd1—O3—C17	−122.9 (3)	C6—N2—C2—C1	−179.7 (3)
N3—Cd1—O3—C17	123.5 (3)	Cd1—N2—C2—C1	−1.2 (3)
N2—Cd1—O3—C17	−39.8 (3)	N1—C1—C2—N2	−33.5 (4)
N1—Cd1—O3—C17	77.8 (3)	N1—C1—C2—C3	148.6 (3)
O1—Cd1—O3—C17	−158.3 (3)	N2—C2—C3—C4	0.6 (5)
O2—Cd1—O4—O4	0.0 (3)	C1—C2—C3—C4	178.4 (3)
N3—Cd1—O4—O4	0.0 (3)	C2—C3—C4—C5	1.0 (5)
N2—Cd1—O4—O4	0.0 (3)	C3—C4—C5—C6	−1.2 (5)
N1—Cd1—O4—O4	0.0 (2)	C2—N2—C6—C5	1.5 (5)
O1—Cd1—O4—O4	0.0 (4)	Cd1—N2—C6—C5	−176.8 (2)
O3—Cd1—O4—O4	0.0 (2)	C4—C5—C6—N2	0.0 (5)
O4—Cd1—O4—C17	0(19)	C13—N1—C7—C8	88.0 (3)
O2—Cd1—O4—C17	44.4 (3)	C1—N1—C7—C8	−150.7 (3)
N3—Cd1—O4—C17	−76.6 (3)	Cd1—N1—C7—C8	−36.2 (3)
N2—Cd1—O4—C17	137.0 (3)	C12—N3—C8—C9	0.0 (4)
N1—Cd1—O4—C17	−155.0 (3)	Cd1—N3—C8—C9	−177.7 (2)
O1—Cd1—O4—C17	38.8 (5)	C12—N3—C8—C7	176.9 (3)
O3—Cd1—O4—C17	−7.5 (3)	Cd1—N3—C8—C7	−0.7 (3)
O4—Cd1—N1—C7	127.65 (19)	N1—C7—C8—N3	26.7 (4)
O4—Cd1—N1—C7	127.65 (19)	N1—C7—C8—C9	−156.3 (3)
O2—Cd1—N1—C7	−77.5 (2)	N3—C8—C9—C10	0.5 (5)
N3—Cd1—N1—C7	26.46 (18)	C7—C8—C9—C10	−176.4 (3)
N2—Cd1—N1—C7	−153.4 (2)	C8—C9—C10—C11	−0.4 (5)
O1—Cd1—N1—C7	−56.62 (19)	C9—C10—C11—C12	−0.1 (5)
O3—Cd1—N1—C7	74.8 (3)	C8—N3—C12—C11	−0.5 (4)
O4—Cd1—N1—C13	3.7 (2)	Cd1—N3—C12—C11	176.9 (2)
O4—Cd1—N1—C13	3.7 (2)	C10—C11—C12—N3	0.6 (5)
O2—Cd1—N1—C13	158.6 (2)	C7—N1—C13—C14	−66.3 (3)
N3—Cd1—N1—C13	−97.5 (2)	C1—N1—C13—C14	171.5 (3)
N2—Cd1—N1—C13	82.7 (2)	Cd1—N1—C13—C14	55.2 (3)
O1—Cd1—N1—C13	179.5 (2)	N1—C13—C14—C14^i^	−173.6 (3)
O3—Cd1—N1—C13	−49.1 (3)	Cd1—O1—C15—O2	6.6 (4)
O4—Cd1—N1—C1	−114.65 (19)	Cd1—O1—C15—C16	−178.5 (4)
O4—Cd1—N1—C1	−114.65 (19)	Cd1—O2—C15—O1	−7.5 (5)
O2—Cd1—N1—C1	40.2 (2)	Cd1—O2—C15—C16	177.4 (4)
N3—Cd1—N1—C1	144.16 (19)	Cd1—O4—C17—O4	0(66)
N2—Cd1—N1—C1	−35.66 (18)	O4—O4—C17—O3	0.00 (9)
O1—Cd1—N1—C1	61.08 (19)	Cd1—O4—C17—O3	15.0 (5)
O3—Cd1—N1—C1	−167.51 (19)	O4—O4—C17—O3	0.00 (9)
O4—Cd1—N2—C2	137.5 (2)	Cd1—O4—C17—O3	15.0 (5)
O4—Cd1—N2—C2	137.5 (2)	O4—O4—C17—C18	0.00 (7)
O2—Cd1—N2—C2	−113.1 (2)	Cd1—O4—C17—C18	−168.0 (4)
N3—Cd1—N2—C2	20.7 (3)	O3—O3—C17—O4	0.00 (10)
N1—Cd1—N2—C2	21.0 (2)	Cd1—O3—C17—O4	−12.0 (4)
O1—Cd1—N2—C2	−60.4 (2)	O3—O3—C17—O4	0.00 (10)
O3—Cd1—N2—C2	171.8 (2)	Cd1—O3—C17—O4	−12.0 (4)
O4—Cd1—N2—C6	−44.1 (3)	Cd1—O3—C17—O3	0(100)
O4—Cd1—N2—C6	−44.1 (3)	O3—O3—C17—C18	0.0 (2)
O2—Cd1—N2—C6	65.3 (3)	Cd1—O3—C17—C18	171.1 (4)

Symmetry codes:  (i) −*x*, −*y*+1, −*z*.

### Hydrogen-bond geometry (Å, °)

**Table:**

*D*—H···*A*	*D*—H	H···*A*	*D*···*A*	*D*—H···*A*
O9—H9A···O3	0.80 (6)	2.06 (6)	2.829 (5)	160 (6)
O6—H6A···O4	0.77 (5)	1.98 (5)	2.745 (4)	170 (5)
O9—H9B···O5^ii^	0.79 (5)	2.07 (5)	2.852 (3)	170 (5)
O7—H7C···O1^iii^	0.76 (5)	1.91 (5)	2.673 (4)	177 (5)
O8—H8A···O9^iv^	0.73 (5)	2.04 (5)	2.769 (4)	172 (5)
O6—H6B···O8^i^	0.85 (5)	1.97 (5)	2.792 (4)	164 (5)
O5—H5A···O7^iii^	0.76 (4)	1.96 (4)	2.708 (3)	170 (5)
O8—H8B···O6^v^	0.78 (5)	2.06 (5)	2.825 (4)	166 (4)

Symmetry codes:  (ii) *x*+1/2, *y*+1/2, *z*; (iii) −*x*+1, *y*, −*z*+1/2; (iv) *x*−1/2, *y*−1/2, *z*; (i) −*x*, −*y*+1, −*z*; (v) *x*, *y*−1, *z*.
